# Supplementary material for: PhosSight: A Unified Deep Learning Framework Boosting and Accelerating Phosphoproteome Identification to Enable Biological Discoveries
Source: Adv Sci (Weinh). 2026 May 27;13(47):e75856. doi: 10.1002/advs.75856 (PMC13335961; doi:10.1002/advs.75856)
Supplement: Supplementary file 1 [file ADVS-13-e75856-s002.docx]

**Supplemental Figures**

**Supplementary Figure 1. PhosDetect model architecture for pre-acquisition phosphopeptide detectability prediction.**

**Supplementary Figure 2. Dataset preparation workflow for PhosDetect model training.**

**Supplementary Figure 3. Cross-species phosphoproteomic benchmarking of PhosDetect, DeepDetect, and PFly using datasets from PeptideAtlas.** A. Bar plot of AUC values for the pre-trained models on three independent phosphoproteomic datasets from rice, mouse, and yeast. B. Bar plot of AUC values after fine-tuning on the same three phosphoproteomic datasets.

**Supplementary Figure 4. Detectability comparison between true detectable phosphoisomers and same-sequence alternative site isomers in the synthetic phosphopeptide benchmark PXD000138.** Violin-box plots show that PhosDetect assigns significantly higher detectability scores to sequence-correct synthetic phosphopeptides with the known ground-truth phosphorylation site (positive) than to the corresponding same-sequence alternative site isomers generated by phosphate re-location.

**Supplementary Figure 5. Site-determining ion analysis of synthetic phosphopeptide PSMs rescued by detectability-assisted re-localization.** A. Pie chart showing the distribution of site-determining ion status among PSMs correctly localized by Method 4 but mislocalized by Method 1. B. Pie chart showing the corresponding distribution among PSMs correctly localized by Method 4 but mislocalized by Method 2. Together, panels A and B show that detectability-assisted re-localization is frequently supported by direct fragment evidence and, in cases lacking site-determining ions, provides additional orthogonal information when fragment evidence alone is insufficient for reliable localization. C-J. Representative spectra from the Method 2 versus Method 4 comparison illustrating the four categories shown in panel B: both methods lacking site-determining ions (C, D), only Method 4 containing site-determining ions (E, F), only Method 2 containing site-determining ions (G, H), and both methods containing site-determining ions (I, J). In panels C-J, False and True indicate incorrect and correct site localization, respectively.

**Supplementary Figure 6. PSM overlap in DIA-NN searches using filtered spectral libraries.** Upset plot displaying the overlap of PSMs across searches using libraries filtered at different proportions based on the fine-tuned PhosDetect model on dataset JPST000859.

**Supplementary Figure 7. Performance comparison of PhosSight against PhosphoRS and DeepRescore2 at the PSM level on two real-world phosphoproteomic datasets, including a label-free dataset and a TMT dataset.** A. The number of identified PSMs from two datasets using five different search engines (Comet, MaxQuant, MS-GF+, X!Tandem, MSFragger) when comparing PhosSight versus PhosphoRS. B. The number of identified PSMs using the same five search engines when comparing PhosSight versus DeepRescore2 on the same two datasets. Gain: identified by PhosSight but not by the other method. Shared: identified by both methods. Loss: identified by the other method but not by PhosSight.

**Supplementary Figure 8. Motif analysis of phosphosites from the training set and from biological datasets localized by Method 1 or PhosSight.** A. Sequence motif of phosphosites in the PhosDetect training dataset. B. Sequence motif of phosphosites identified in the label-free dataset and localized by Method 1. C. Sequence motif of phosphosites identified in the label-free dataset and localized by PhosSight. D. Sequence motif of phosphosites identified in the TMT dataset and localized by Method 1. E. Sequence motif of phosphosites identified in the TMT dataset and localized by PhosSight. For all panels, phosphosites were centered within 15-mer sequences with seven flanking amino acids on each side. The overall motif patterns remained highly similar between Method 1 and PhosSight in both biological datasets, arguing against an obvious shift of re-localized sites toward training-set motif preferences.

**Supplementary Figure 9. Phosphopeptide overlap across DIA-NN searches using filtered spectral libraries.** Upset plot displaying the overlap of phosphopeptides identified by DIA-NN searches with spectral libraries filtered at different proportions based on the fine-tuned PhosDetect model. The analysis was conducted on A549 DIA phosphoproteomics data.

**Supplementary Figure 10. Phosphopeptide-specific PSM overlap across DIA-NN searches using filtered spectral libraries.** Upset plot displaying the overlap of phosphopeptide-specific peptide-spectrum matches (PSMs) identified by DIA-NN searches with spectral libraries filtered at different proportions based on the fine-tuned PhosDetect model. The analysis was conducted on A549 DIA phosphoproteomics data.

**Supplementary Figure 11. Comparative analysis of the overlap between the fine-tuned PhosDetect-filtered library and the original unfiltered library at the phosphopeptide and PSM levels.** A. Overlap of phosphopeptides identified from searches using the fine-tuned PhosDetect model with top 50% detectability filtering and the original unfiltered library. B. Overlap of all PSMs identified from the two searches. C. Overlap of phosphopeptide-specific PSMs identified from the two searches. Detectability (D) and delta RT (E) distributions across phosphopeptide subsets from (A).
